# Rethinking “Exercise is Medicine”

**DOI:** 10.17179/excli2020-2613

**Published:** 2020-08-18

**Authors:** Shunchang Li, Ismail Laher

**Affiliations:** 1 Institute of Sports Medicine and Health, Chengdu Sport Institute, Chengdu, 610041, China; 2 Faculty of Medicine, Department of Anesthesiology, Pharmacology and Therapeutics, The University of British Columbia, Vancouver, BC, Canada

## ⁯

***Dear Editor,***

"Exercise is Medicine" (EiM) is a United States-based health initiative that was co-launched by the American College of Sports Medicine (ACSM) and the American Medical Association (AMA) in 2007. Public health and research institutions in several countries and regions have embraced this philosophy, and over 40 countries have embraced EiM as a health initiative. EiM focuses on making exercise in the promotion of health care (ACSM, 2020[1]). Undoubtedly, without strong epidemiological and medical evidence, the promotion of physical activity will limit motivation (WHO, 2018[8]). However, there are many views on the nature and purpose of exercise, suggesting that sports medicine and exercise scientists should perhaps rethink whether EiM is an effective means of promoting physical activity?

### Logic of exercise health promotion is self-limiting. 

The logic behind EiM recognizes that non-communicable diseases (such as diabetes, cardiovascular diseases, obesity etc.) are closely related to sedentary lifestyles (Segar et al., 2016[7]). Exercise intervention is an extension and derivative of medical treatment and health care. Several studies confirm that acute exercise improves cognitive ability, reduces anxiety, and increases well-being; long-term regular exercise can control weight, reduce the risk of cardiovascular diseases, delay the onset of type 2 diabetes, enhance bone and muscle mass, prevent falls and prolong life in the elderly (Lobelo et al., 2014[4]). Thus, as for the people at high risk of non-communicable chronic diseases, exercise can be used as a "vaccine" to reduce the risk of disease and improve the quality of life. However, not all non-communicable diseases are caused by sedentary lifestyles. For example, although most cardiovascular diseases develop in adulthood, the causative factors can often be traced to adolescence and even to the embryonic period (Chiesa et al., 2019[3]). In this case, the beneficial or harmful effects of exercise intervention depend on the specific condition of the diverse diseases, and exercise is not suitable for all non-communicable chronic diseases, such as spinal cord injury and arthritis. In addition, there is no evidence that exercise combats infectious diseases such as SARS, MESR and COVID-19. 

### The purpose of medicine is "to seek common ground", and the essence of sports is "to chase the threshold". 

The goal of all medical treatments is to cure abnormal physical and mental condition, and to restore physiological indicators normal or near-normal values. In contrast, exercise applies an optimal stimulus to the body to promote better health and well-being. Acute exercise causes physiological indicators (such as heart rate, temperature, etc.) to gradually approach boundary values by swaying from average levels; chronic endurance exercise training induces a series of adaptive changes in the cardiovascular system, such as exercise-induced sinus bradycardia and exercise-induced cardiac hypertrophy. These are accepted as normal "exercise-induced adaptations" but are considered to be "abnormal phenomena" in clinical medicine. Moreover, long-term resistance exercise training increases muscle mass and dimensions, so that the larger body mass index (BMI) is not clinically classified as “obese”.

### Medicine restores health, exercise promotes health. 

EiM requires positive feedback of health-related physiological indicators (such as weight, blood pressure, blood sugar, blood lipids, etc.) to maintain exercise compliance. However, changes in physiological indicators often require a period of adaptation and accumulation, and there are obvious individual differences due to some non-motor factors such as ethnicity, genetics, gender, and physical activity levels (Rawlins et al., 2010[6]; Mao et al., 2020[5]; Bouchard et al.,, 2011[2]). Delayed or negative feedback will greatly weaken the adherence of continuous exercise and reduce exercise compliance. Moreover, EiM weakens or ignores the role of exercise in promoting physical and mental health. Exercise is a manifestation of our inherent survival instincts, increasing our prowess in the hunter-gatherer environment by improving our agility and strength. 

**Figure 1:**
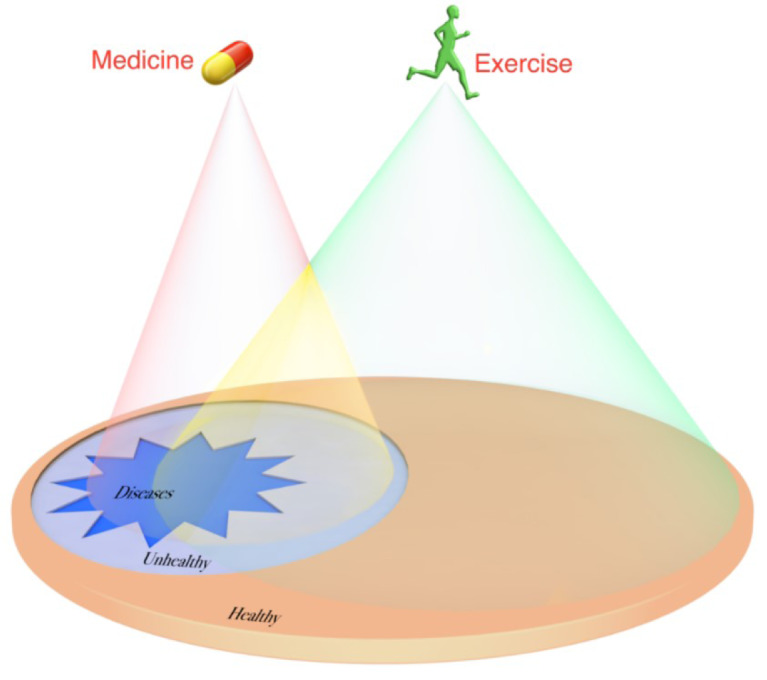
Exercise Intervention and Medicine Treatment. Although these two interventions (exercise and medicine) overlap, each has its own scope of application. *Note: Exercise intervention in green, medical treatment in purple, and overlap of exercise intervention and medical treatment in yellow.*

Good health enables people to complete tasks and also realize self-worth by maintaining energy and vitality. Thus, exercise is more than medicine. A series of randomized controlled trails on workers where exercise intervention protocols were personalized based on the principles of exercise science, occupational exposure, health status and exercise capacity of workers reported that exercise interventions during work significantly reduced health risk indicators and improved exercise capacity and also decreased sickness and absenteeism while also improving productivity and work efficiency (Lobelo et al., 2014[4]). See also Figure 1(Fig. 1).

### Summary

Exercise is Medicine (EiM), which uses medicine as a logical starting point, actively promotes the health and social aspects of physical activity, and counteracts the health risks of a sedentary lifestyle. However, EiM rarely considers many important aspects of exercise science. We need to acknowledge that exercise and medicine are both important components of maintaining and promoting health, but the blanket statement that “exercise is medicine” maybe misleading as some people engage in unscientific and unsuitable physical exercise, and also causing sports and exercise scientists to deviate from exploring the various physiological components of exercise.

## Conflict of interest

The authors declare no conflict of interest.
